# Development and Validation of a Clinical Pregnancy Failure Prediction Model for Poor Ovarian Responders During IVF/ICSI

**DOI:** 10.3389/fendo.2021.717288

**Published:** 2021-08-23

**Authors:** Fangyuan Li, Ruihui Lu, Cheng Zeng, Xin Li, Qing Xue

**Affiliations:** Department of Gynecology and Obstetrics, Peking University First Hospital, Beijing, China

**Keywords:** predictive model, clinical pregnancy failure, poor ovarian response, IVF/ICSI, nomogram

## Abstract

**Backgrounds:**

Despite the great advances in assisted reproductive technology (ART), poor ovarian response (POR) is still one of the most challenging tasks in reproductive medicine. This predictive model we developed aims to predict the individual probability of clinical pregnancy failure for poor ovarian responders (PORs) under *in vitro* fertilization/intracytoplasmic sperm injection (IVF/ICSI).

**Methods:**

The nomogram was developed in 281 patients with POR according to the Bologna criteria from January 2016 to December 2019, with 179 in the training group and 102 in the validation group. Univariate and multivariate logistic regression analyses were used to identify characteristics that were associated with clinical pregnancy failure. The nomogram was constructed based on regression coefficients. Performance was evaluated using both calibration and discrimination.

**Results:**

Age >35 years, body mass index (BMI) >24 kg/m^2^, basic follicle-stimulating hormone (FSH) >10 mIU/ml, basic E2 >60 pg/ml, type B or C of endometrium on human chorionic gonadotropin (hCG) day, and the number of high-quality embryos <2 were associated with pregnancy failure of POR patients. The area under the receiver operating characteristic curve (AUC) of the training set is 0.786 (95% confidence interval (CI): 0.710–0.861), and AUC in the validation set is 0.748 (95% CI: 0.668–0.827), showing a satisfactory goodness of fit and discrimination ability in this nomogram.

**Conclusion:**

Our nomogram can predict the probability of clinical pregnancy failure in PORs before embryo transfer in IVF/ICSI procedure, to help practitioners make appropriate clinical decisions and to help infertile couples manage their expectations.

## Introduction

With the development of *in vitro* fertilization/intracytoplasmic sperm injection (IVF/ICSI) techniques over the past decades, the purpose of personalized treatment of IVF/ICSI is to help every couple maximize the chances of pregnancy and eliminate the avoidable risks resulting from ovarian stimulation (1). Despite the advances in assisted reproductive technology (ART), poor ovarian response (POR) poses a great challenge in that the number of oocytes collected is usually below expectation with the appropriate ovarian stimulation (2–4), leading to fewer transferable embryos, greater odds for cycle cancellation, and lower pregnancy rates (as low as 2%–4%) (5–7). The incidence of poor ovarian responders (PORs) among infertile women has been reported to vary between 5.6% and 35.1% (8–11), and POR affects approximately 11.9% women in China undergoing IVF treatment (12). Therefore, POR is considered as one of the success-limiting factors for IVF/ICSI outcomes (13).

As ART treatment is expensive, invasive, and not a guarantee of success, infertile couples need to be informed about their chances of pregnancy to manage their expectations. Although some studies have used multivariate regression models to identify predictive factors associated with IVF/ICSI outcomes (14–16), none of them targeted evaluating the chances of clinical pregnancy in PORs.

Therefore, we aimed to evaluate the risk of poor pregnancy outcomes and develop a nomogram to predict the probability of clinical pregnancy failure in patients with POR before embryo transfer in IVF/ICSI procedure, in order to make appropriate clinical decisions and help couples manage their expectations.

## Materials and Methods

### Patients’ Selection

Patients who underwent IVF/ICSI–embryo transfer (IVF/ICSI–ET) cycles between January 2016 and December 2019 were retrospectively reviewed in the Department of Obstetrics and Gynecology, Peking University First Hospital. All women were routinely requested for their data to be used for research purposes, and those who refused consent were excluded from this study. The local ethics committee granted permission to this study.

Patients were eligible if they fulfilled the definition of POR according to the Bologna criteria (17)—at least two of the following three features must be present: 1) advanced maternal age (≥40 years) or any other risk factors for POR (evidence of ovarian cysts, previous ovarian surgery, previous chemotherapy, and shortening of the menstrual cycle); 2) a previous POR cycle (≤3 oocytes retrieved or a previous cycle canceled because of ≤3 developing follicles with a conventional stimulation protocol using at least 150 IU follicle-stimulating hormone (FSH) per day); 3) decreased ovarian reserve [i.e., antral follicle count (AFC) <7 follicles or anti-Müllerian hormone (AMH) <1.1 ng/ml].

The exclusion criteria were as follows: hydrosalpinx, uterine fibroids, adenomyosis, uterine malformations, intrauterine adhesion, recurrent spontaneous abortion, antiphospholipid syndrome, chromosome karyotype abnormality, drug allergies, mental disorders and disturbance of consciousness, and a total number of previous IVF cycles >3.

Finally, a total of 281 patients were included in this study. They were included only for one cycle with transfer of two fresh embryos (the first cycle after fulfilling the criteria).

### Controlled Ovarian Hyperstimulation and Embryo Transfer

A long gonadotrophin (Gn)-releasing hormone (GnRH) agonist (GnRH-a) protocol, an antagonist protocol, or a mild ovarian stimulation protocol was used for ovarian stimulation in this study.

The GnRH-a protocol consisted of daily injections of short-acting and long-acting GnRH-a at different doses during the early follicular or mid-luteal phases. For the daily short-acting GnRH-a injections, patients received an injection of 0.1 mg/day of Decapeptyl (Ferring AG, Dübendorf, Switzerland) from the mid-luteal phase of the previous cycle and continued for approximately 15 to 18 days. After ovarian suppression, the dose of Decapeptyl was reduced to 0.05  mg/day and continued until the day of hCG (Zhuhai Lizhu Pharmaceutical Co., Ltd., Zhuhai, China) administration. For the administration of long-acting GnRH-a protocols, triptorelin (Ipsen Pharma Biotech, Signes, France) was injected during the early follicular period, and Gn was injected after 21 to 35 days.

The GnRH antagonist protocol consisted of daily Gn stimulation from days 2 to 3 of menstruation, followed by daily injections of 0.25 mg of Cetrotide (Baxter Oncology GmbH, Frankfurt, Germany) once the leading follicle reached 14 mm and until the day of hCG injection.

Regarding the mild ovarian stimulation, GnRH antagonist was added when the dominant follicle reached 14 mm and until the day of hCG injection.

The choice of protocol for ovarian stimulation was based on the patient’s characteristics. When more than three leading follicles measured 18 mm or more, hCG was administered. Thirty-six hours later, oocyte retrieval was performed under ultrasonic guidance followed by IVF/ICSI. Embryos were transferred on day 2 or 3. The luteal phase was supported by daily vaginal or intramuscular progesterone until 2 weeks after ET.

### Data Collection

Patient clinical parameters (age, stimulation protocols, body mass index (BMI), type of infertility, hormones concentration on day 3 and on hCG day, Gn dose, type and thickness of endometrium, and clinical outcomes) were collected from our database according to the literature review and clinical experiences. Metaphase II (MII) oocytes were determined 16–18 h following retrieval for conventional IVF/ICSI cycles.

Endometrial features including endometrial thickness and pattern were assessed on the day of hCG administration under B‐ultrasonography. Endometrial thickness was measured in a median longitudinal plane of the uterus as the maximum distance between the endometrial–myometrial interface of the anterior to the posterior wall of the uterus. The endometrial pattern was classified as pattern A (a triple-line pattern consisting of a central hyperechoic line surrounded by two hypoechoic layers), pattern B (an intermediate pattern with the same reflectivity as the surrounding myometrium and a poorly defined central echogenic line), and pattern C (homogenous, hyperechogenic endometrium).

Cleavage embryos were classified as high-quality embryos (grade I and II embryos) if they had three to five cells on day 2 or seven to nine cells on day 3 and as same-sized blastomeres if with less than 20% blastomeric fragments. Embryos graded III or IV including those that had only two cells on day 2, less than seven cells on day 3, and no less than 20% fragmentation were called poor quality.

Clinical pregnancy was established by gestational sacs and embryo buds under B‐ultrasonography 4 weeks after embryo transplantation.

### Statistical Analysis

Patients were divided into a training set and a validation set by the sampling techniques of random numbers. Baseline characteristics of patients were expressed as descriptive statistics. Continuous variables are shown as mean ± standard deviation (SD) (normally distributed) and median (interquartile range) (non-normally distributed). Student’s t-tests (normally distributed) or the Mann–Whitney U-test (non-normally distributed) were used to compare variables between groups. Categorical variables are presented as percent, and the chi-squared test was used for statistical comparison of percentages. These data were analyzed with SPSS 22.0.

A univariate logistic regression analysis was used to identify predictors associated with adverse maternal outcomes. The cutoff points of candidate variables were chosen to develop the model based on clinical availability. Age >35 years is used to diagnose advanced maternal age. BMI greater than 24 kg/m^2^ can be diagnosed as overweight according to the Chinese standards (18). Duration of infertility >3 years is considered to be associated with low pregnancy rates (19). We chose some indicators of the diminished ovarian reserve (DOR) to explore whether they could predict the clinical pregnancy in a model such as basal FSH over 10 mIU/ml, basal FSH/basal luteinizing hormone (LH) >3, basal E2 >60 pg/ml, AMH level <0.7 ng/ml, and the number of AFC ≤5 (17, 20–22). Endometrial thickness >7 mm and triple-line pattern are endometrial receptivity markers as prognostic factors for conceiving (23). A meta-analysis of over 60,000 fresh IVF cycles showed a decreased probability of pregnancy achievement in women with progesterone elevation (PE) on the day of hCG administration (when PE was defined using a threshold >0.8 ng/ml) compared with those without PE (24). The other cutoff points for the number of retrieved oocytes, MII oocytes, and high-quality embryos were based on receiver operating characteristic (ROC) analysis. We used Youden’s index (25) to calculate the optimal cutoff points of the three parameters related to the occurrence of pregnancy failure. A multivariate logistic regression analysis was performed to test the independent significance of different factors. The variables with p-values <0.1 in univariate analysis were included in multivariate analysis. The variables were selected by stepwise regression and then fit a more parsimonious model. Variables entered into the model were age, BMI, basal E2, basal FSH, type of endometrium on human chorionic gonadotropin (hCG) day, and the number of high-quality embryos.

The area under the ROC curve (AUC) was used to evaluate the predictive accuracy. Calibration curves were assessed graphically by plotting the observed rates against the predicted probabilities to evaluate the agreement. The Brier score was used to evaluate probability calibration. Nomograms are a pictorial representation of a complex mathematical formula that uses two or more known variables to calculate an outcome. The resulting model was simplified into a nomogram to predict the possibility of clinical pregnancy failure for PORs.

The performance of the nomogram was quantified concerning discriminative power and calibration in the validation cohort for external validation. An internal validation step was performed to counteract the possible overfitting of our model to the data. The bootstrap (with 200 bootstrapped samples) was used to validate and correct the over‐optimism of the models. We also did a decision-curve analysis to assess the clinical applicability of the model.

All analyses were performed using the R software, version 3.6.1.

## Results

### Description of the Study Population

After the inclusion and exclusion criteria of the current study were applied, a total of 281 POR patients who underwent the IVF/ICSI procedures from January 2016 to December 2019 were identified as eligible and were analyzed in this study. Then, the patients were divided into the training set (n = 179) to build the model, and the validation set (n = 102) to test the performance. The basic characteristics are summarized in **Table 1**. Except for the Gn days (p = 0.040), no significant difference is observed in the baseline characteristics between the two groups. Sixty-one patients (34.08%) achieved clinical pregnancy in the training set.

**Table 1:** Basic characteristics of PORs in the training and validation cohorts.

Characteristics	Training set (n = 179)	Validation set (n = 102)	p-Value
Age (years)	38 (34–41)	37 (33–41)	0.054
Stimulation protocols			0.197
Pituitary downregulation (%)	35.75 (64/179)	44.12 (45/102)	
Non-pituitary downregulation (%)	64.25 (115/179)	56.88 (57/102)	
BMI (kg/m^2^)	22.46 (20.19–24.46)	21.87 (19.83–21.15)	0.182
Type of infertility			0.633
Primary infertility (%)	40.22 (72/179)	43.14 (44/102)	
Secondary infertility (%)	59.78 (107/179)	56.68 (58/102)	
Duration of infertility (years)	3 (1–5)	3 (2–5)	0.958
Basal FSH (mIU/ml)	8.59 (7.08–10.77)	8.48 (7.05–9.75)	0.085
Basal FSH/basal LH	2.50 ± 1.00	2.31 ± 0.85	0.093
Basal E2 (pg/ml)	41 (29–57)	44 (33–57)	0.686
AMH (ng/ml)	0.83 (0.54–1.23)	0.89 (0.56–1.06)	0.400
E2 on hCG day (pg/ml)	1,563 (912–2,450)	1,851 (1,228–2,605)	0.058
LH on hCG day (mIU/ml)	1.82 (1.13–2.85)	2.12 (1.36–3.27)	0.162
P on hCG day (ng/ml)	0.84 (0.60–1.53)	0.92 (0.69–1.36)	0. 070
AFC (n)	6 (4–7)	6 (5–7)	0.664
Gonadotropin dose (IU)	2,960.75 ± 1,099.84	3,150.37 ± 942.36	0.145
Gonadotropin days (days)	9 (8–11)	10 (9–12)	0.040*
Endometrial thickness (mm)	10 (9–12)	10 (9–12)	0.7077
Type of endometrium			0.667
Type A (%)	37.43 (67/179)	34.31 (35/102)	
Type B or C (%)	62.57 (112/179)	65.05 (67/102%)	
Oocytes retrieved (n)	4 (2–7)	5 (4–7)	0.054
MII oocytes (n)	3 (2–6)	4 (3–6)	0.051
High-quality embryos (n)	1 (1–3)	2 (1–3)	0.157
Clinical pregnancy (%)	34.08 (61/179)	43.14 (44/102)	0.131

Pituitary downregulation means GnRH agonist long protocol. Non-pituitary downregulation includes GnRH antagonist protocol and the mild ovarian stimulation protocol. Continuous variables are shown as the median (interquartile range) or mean ± standard deviation. Categorical variables are presented as percent.

BMI, body mass index; FSH, follicle-stimulating hormone; LH, luteinizing hormone; P, progesterone; E2, estradiol; AMH, anti-Müllerian hormone; hCG, human chorionic gonadotrophin; hCG E2, hCG LH, or hCG P means E2, LH, or P on the day of hCG administration; AFC, antral follicle count; endometrial thickness, the endometrial thickness on the day of hCG injection.

*Training set vs. validation set: p < 0.05.

### Logistic Regression Analysis

The univariate logistic regression analysis of pregnancy failure in the developing group is listed in **Table 2**. The optimal cutoff points of the values were chosen according to the clinical consensus or ROC curve of our data. According to the univariate logistic regression analysis, values with p < 0.1 were included in the multivariate logistic regression analysis for pregnancy failure.

**Table 2:** Univariate analysis in the training group.

Variables	OR (95% CI)	p-Value
Age > 35 (years)	3.77 (1.92–7.53)	<0.001
Non-pituitary downregulation protocol	1.28 (0.65–2.48)	0.469
BMI > 24 (kg/m^2^)	2.21 (1.12–4.52)	0.025
Secondary infertility	1.26 (0.65–2.43)	0.485
Duration of infertility > 3 (years)	1.37 (0.71–2.70)	0.353
Basal FSH > 10 (mIU/ml)	2.07 (1.04–4.31)	0.043
Basal FSH/basal LH > 3	0.658 (0.34–1.31)	0.225
Basal E2 > 60 (pg/ml)	2.03 (0.99–4.38)	0.061
AMH < 0.7 (ng/ml)	1.76 (0.87–3.74)	0.125
P on hCG day > 0.8 (ng/ml)	0.71 (0.37–1.36)	0.305
AFC ≤ 5 (n)	0.58 (0.30–1.11)	0.103
Endometrial thickness ≤ 7 (mm)	1.11 (0.43–3.24)	0.845
Type B or C of endometrium	2.73 (1.41–5.35)	0.003
Oocytes retrieved ≤ 3 (n)	1.61 (0.82–3.26)	0.173
MII oocytes ≤ 3 (n)	1.78 (0.93–3.46)	0.082
High-quality embryos < 2 (n)	2.26 (1.15–4.45)	0.018

OR, odds ratio; CI, confidence interval; BMI, body mass index; FSH, follicle-stimulating hormone; AMH, anti-Müllerian hormone; hCG, human chorionic gonadotrophin; AFC, antral follicle count; MII, metaphase II; LH, luteinizing hormone.

### Development of the Models From the Training Cohort

The variables were selected by stepwise regression and then fit a more parsimonious model. Finally, as shown in **Table 3**, the six independent risk factors for clinical pregnancy included in the prediction model are as follows [OR (95% CI), p-value]: age >35 years [2.59 (1.24–5.47), p = 0.012], BMI >24 kg/m^2^ [3.22 (1.45–7.58), p = 0.005], basic FSH >10 mIU/ml [2.87 (1.28–6.75), p = 0.012], basic E2 >60 pg/ml [2.47 (1.08–5.93), p = 0.036], type B or C of endometrium on hCG day [2.47 (1.18–5.24), p = 0.017], and the number of high-quality embryos <2 [2.24 (1.02–4.96), p = 0.045].

**Table 3:** Multivariate logistic regression model in the training set.

Variables	Regression coefficients	OR (95% CI)	p-Value
Age > 35 (years)	0.953	2.59 (1.24–5.47)	0.012
BMI > 24 (kg/m^2^)	1.169	3.22 (1.45–7.58)	0.005
Basal FSH > 10 (mIU/ml)	1.053	2.87 (1.28–6.75)	0.012
Basal E2 > 60 (pg/ml)	0.902	2.47 (1.08–5.93)	0.036
Type B or C of endometrium	0.906	2.47 (1.18–5.24)	0.017
High-quality embryos < 2 (n)	0.806	2.24 (1.02–4.96)	0.045

OR, odds ratio; CI, confidence interval; BMI, body mass index; FSH, follicle-stimulating hormone.

**Figure 1:**
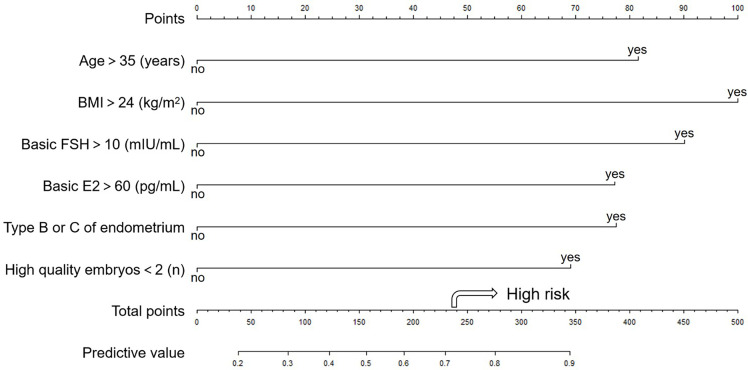
The nomogram to predict the probability of clinical pregnancy failure in PORs. The nomogram can be applied by following procedures: (A) draw a line perpendicular from the corresponding axis of each risk factor until it reaches the top line labeled “Points”; (B) sum up the points for all risk factors and recorded as the total score; and (C) draw a line descending from the axis labeled “Total points” until it intercepts the lower line to determine the probability of failed conception. The arrow shows high risk when the total points are greater than 236.472. The optimal threshold point was calculated using receiver operating characteristic (ROC) curve.

The nomogram of prediction is shown in **Figure 1**. The optimal threshold point was calculated using the ROC curve. When the total points are greater than 236.472, women with POR show a high risk for a failed pregnancy.

### Model Validation

**Figure 2:**
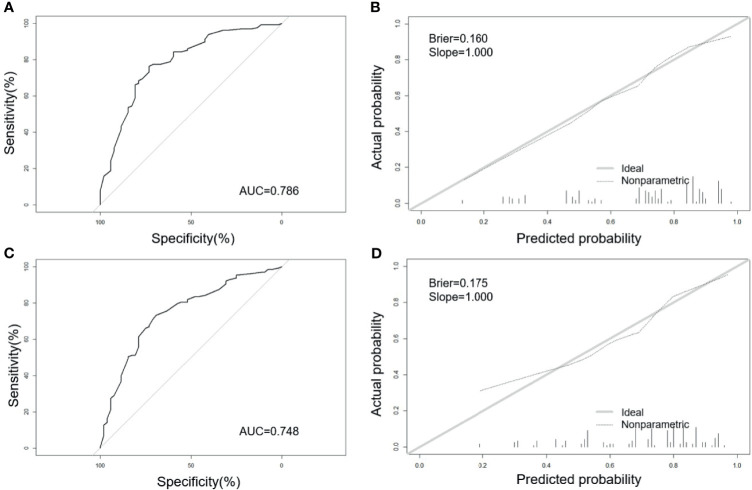
Receiver operating characteristic (ROC) curves and calibration plots of the training and validation sets. **(A)** Area under the ROC curve (AUC) of the training set is 0.786 (95% CI: 0.710–0.861). **(B)** Calibration curve for training set (Brier = 0.160, Slope = 1.000). **(C)** AUC of the validation set is 0.748 (95% CI: 0.668–0.827). **(D)** Calibration curve for validation set (Brier = 0.175, Slope = 1.000). Calibration curves were used to evaluate the calibration of the model. The horizontal axis is the predicted probability provided by this model, and the vertical axis is the observed incidence of pregnancy failure. The ideal line with 45° slope represents a perfect prediction (the predicted probability equals the observed probability). The lower the Brier score for a set of predictions, the better the prediction calibration. When the slope was closer to 1.00, the prediction model had better calibration power.

The AUC of the model in the training set (**Figure 2A**) is 0.786 (95% CI: 0.710–0.861), which indicated a good performance. The sensitivity is 73.1%, and the specificity is 76.4%. The AUC of the model in the validation set (**Figure 2C**) is 0.748 (95% CI: 0.668–0.827). The sensitivity is 69.2%, and the specificity is 73.2%. The slope of calibration curves in the training set (**Figure 2B**) and the validation set (**Figure 2D**) is 1.000 and 1.000, respectively. The predictive model has better calibration power when the slope is closer to 1.000. The Brier score of calibration curves in the training set and validation set is 0.160 and 0.175, respectively, which shows that the model is well-calibrated.

Internal validation (**Table 4**) shows performance indices of the model corrected for optimism after 200 bootstrapped samples. Overall, the predictive model performs well, even after correction for optimism.

**Table 4:** Performance of internal validation.

Index	Original	Optimism	Optimism-corrected value
Dxy	0.571	0.051	0.520
R^2^	0.281	0.054	0.228
Brier	0.160	−0.013	0.173

The original dataset was corrected for optimism with 200 bootstrap samples.

The decision-curve analysis shows that the prediction model is the higher line on the decision curve, which indicates that the prediction model leads to a higher net benefit and greater clinical utility (**Supplementary 1**).

## Discussion

This predictive model we developed aims to predict the individual probability of failed clinical pregnancy for women with POR under IVF/ICSI-ET. The nomogram was developed in a training cohort including 179 PORs and tested on an external independent validation cohort including 102 patients with POR. Performance was evaluated using both calibration and discrimination. We have established three models at different time points for pregnancy failure prediction: model A (before ET), model B (before the start of IVF/ICSI cycle), and model C (on hCG day) (**Supplementary 2, 3**). After multiple comparisons, we chose the best-performing model A as our final prediction nomogram. Our nomogram is a user-friendly graphical representation of the model. The covariates of our model depend on the combination of readily available clinical and biological characteristics including patient age, BMI, basal E2, basal FSH, type of endometrium on hCG day, and the number of high-quality embryos, which are clinically significant and concordant with the published data.

There are a few models for predicting the success of clinical pregnancy of patients undergoing IVF/ICSI procedure in recent years (15, 16, 26). In a pregnancy prediction model based on 1,675 IVF–double fresh embryo transfer cycles, there were no internal or external validations, and the predictive ability was relatively poor (26). A nomogram with good performance (AUC: 0.76) to predict the clinical pregnancy rate was only based on patients with endometriosis (16). Another model found independent predictors of the chance of clinical pregnancy after a completed IVF/ICSI cycle and did not focus on the special population of POR (15). Therefore, the advantages of our predictive model are as follows: first, it is a complete nomogram using a stepwise regression method with internal and external validations; second, the performance is quite good (AUC of 0.786); last but not least, it focuses on PORs, which is one of the high-risk groups associated with poor clinical pregnancy in IVF/ICSI cycles. We hypothesize that this nomogram can be used in the routine practice to facilitate physicians in predicting the pregnancy rate of PORs, selecting more appropriate individualized treatments, and helping patients with POR manage their expectations for conception.

In our model, maternal age >35 years is an independent risk factor of pregnancy failure. Patient age has been reported to be a vital prognostic factor in reproductive medicine and is frequently involved in evaluating the probability of pregnancy. Increased patient age is associated with decreased clinical pregnancy rate (16, 27, 28). In an Australian cohort of 36,412 patients initiated with first autologous fresh IVF cycles, for women ≥30 years, every 1-year increase in age was associated with an 11% reduction in the chance of achieving pregnancy. If women aged 35 years or older would have had their first autologous fresh treatment 1 year earlier, 15% extra deliveries would be expected (29). The trend that older poor responders have a lower pregnancy rate compared with younger poor responders has been revealed in several researches (10, 12, 30, 31). For poor responders over 35 years, the rates of implantation and clinical pregnancy were lower than those of under 35. Meanwhile, younger poor responders (<35) still have a reasonable number of transferable embryos (2.02 ± 0.57) and an acceptable pregnancy rate (37.50%) (32). It is well understood that increased age leads to a reduction in the quantity and quality of oocytes, which is accompanied by a decline in female fertility (33, 34). Therefore, PORs should be treated positively, and IVF/ICSI treatment should be considered earlier.

Overweight and obesity raise major challenges for women of reproductive age. Up to 60% of worldwide women are overweight, and up to 30% of these women are obese (35, 36). In a large retrospective study of 500,000 autologous IVF cycles, obese women had a 6% reduction in intrauterine pregnancy rates and a 13% reduction in live birth rates as compared with normal-weight women (37). Obese poor responders might have a lower pregnancy rate than non-obese poor responders. One study described a significant decrease in pregnancy rate for the poor responders with BMI >30 kg/m^2^ versus BMI ≤30 kg/m^2^ (4.5% versus 23%, respectively). However, non-obese PORs achieved pregnancy rates comparable with those of normal responders (38). Correlation analysis in our cohort revealed that both overweight status and obese status of PORs were related to receiving pregnancy failure and could be a predictor for adverse pregnancy outcomes. This is because impaired ovarian follicular genesis (39), oocyte quality (40, 41), embryonic development (40, 42), and endometrial receptivity (41, 43) might be involved in poorer reproductive outcomes in obese women. Fortunately, high body weight is a reversible basic parameter. Weight loss of 10 kg over 6 months was shown to improve ovulation function and rates of conception in obese anovulatory women (44). Therefore, due to the high risk of low pregnancy rates in PORs, weight management should be encouraged for them in preconception counseling to potentially improve ART outcomes as well as reduce Gn dosage and anesthetic dosage during oocyte retrieval.

Our data indicated that increasing basal FSH levels were associated with lower pregnancy rates in women with POR. With a similar cutoff point, two studies found that the chance of pregnancy was significantly higher in women with basal FSH <10 IU/L than in women with FSH ≥10 IU/L under IVF treatment (26, 45). In an analysis of 163 poor responders, the pregnancy rate for patients with an elevated basal FSH (>12.0 IU/L) was significantly decreased versus those with normal FSH (4.0% versus 14.8%, respectively) (30). As is well known, the FSH level shows a rising trend with increasing age. Some researchers hold an interesting point that younger ages seem to protect a woman against the negative effect of a raised FSH concentration (45, 46). This further suggests that PORs require ART as soon as possible to have more chance of conceiving, since ovarian responsiveness and clinical outcome deteriorate with increasing age and serum FSH. Meanwhile, basal serum FSH >10 IU/L indicates the DOR, which is closely related with the number and quality of retrieved oocytes. In this regard, the importance of basal FSH concentration would lie in developing a tailored stimulation protocol that can maximize the ovarian response by achieving a successful pregnancy outcome, especially in PORs. However, some other indicators of DOR (such as AMH and AFC) were not specific to screen for failure to conceive for PORs in our data. There is emerging evidence to support our findings that the low AMH cutoff points (0.2–0.7 ng/ml) or low AFC (≤5) have moderate-to-high specificity as a screening test for POR but not specific for predicting pregnancy failure (47–50). Also, AMH and AFC are relatively low in a majority group of PORs, which may reduce the prediction performance.

An early rise in serum basal E2 concentration is a classic feature of reproductive aging. Baseline E2 level >60 pg/ml is used as a risky predictor for pregnancy failure in our model. When the basal FSH concentration is normal but the serum estradiol level is above 60–80 pg/ml in the early follicular phase, there is some evidence of association with poor response, increased cancellation rates, and decreased pregnancy rates (51–53). We speculate that there are two reasonable explanations. On the one hand, DOR is responsible for poor pregnancy outcome. The diminished inhibin production from the pituitary increases basal FSH level and secondarily increases E2 production from granulosa cells in the ovary in the early follicular phase of women with decreased ovarian reserve and function. This in turn will suppress FSH production and release. A temporary balance between pituitary and ovarian response will result in normal FSH levels and increased circulating E2. On the other hand, the limited number of oocytes retrieved causes low pregnancy rates. High basal E2 hinders the development of the dominant follicle, which influences the ovarian response and decreases the total number of retrieved oocytes after stimulation.

In IVF/ICSI fresh ET cycles, high-quality embryos will be preferentially transferred into the uterine cavity because good quality is associated with high success rate of clinical pregnancy. In our model, the number of high-quality embryos below 2 boosts pregnancy failure risk. It is comprehensible that in the two transferred embryos per fresh cycle, both of them belong to grade I or II and will be more likely to implant into the endometrium. Therefore, women with POR take greater risks of transfer cancellation to accumulate good embryos. The endometrial pattern also predicts the pregnancy outcome in our model. Pattern A with complete triple line at ultrasound examination reflects endometrial proliferation. This presence on the day of hCG injection is associated with a higher pregnancy rate than the absence of this pattern (54), which is consistent with our conclusion. However, whether endometrial thickness affects pregnancy is controversial. One study summarized that similar clinical pregnancy rates were found between women with triple-line pattern and women without triple-line pattern assessed on the day of hCG undergoing IVF with fresh ET (55). The absence of a triple-line pattern may be a sign of premature secretory changes of the endometrium and the passed time-window of maximal endometrial receptivity (56). This status is not conducive to embryo implantation. However, whether endometrial thickness affects pregnancy is controversial. One study summarized that similar clinical pregnancy rates were found between women with triple-line pattern and women without triple-line pattern assessed on the day of hCG undergoing IVF with fresh ET (55). Further data relevant to the clinical value of the endometrial patterns are needed.

The main limitation of our model is its retrospective nature, which cannot exclude all potential biases. Besides, the data collection is based on our single center, and no independent external validation cohorts from other hospitals were included in our study. Furthermore, due to the high cancellation rates of PORs, further studies concerning the predictive factors for cumulative pregnancy after fresh and frozen-thawed ET cycles are needed. Prospective, large-scale, and multicenter clinical trials should be carried out in the future.

## Conclusion

In conclusion, our analysis resulted in a well-calibrated model that can predict the risk of clinical pregnancy failure in PORs under IVF/ICSI-ET cycles to help physicians choose more appropriate individualized treatments and to help patients with POR to manage their expectations for ART outcomes.

## Data Availability Statement

The datasets presented in this article are not readily available because This database relates to the confidentiality of our clinical center and patient privacy, so it is not convenient to disclose. Requests to access the datasets should be directed to FL, lfyhelen24@163.com.

## Ethics Statement

The studies involving human participants were reviewed and approved by Biomedical Research Ethics Committee of Peking University First Hospital. The patients/participants provided their written informed consent to participate in this study.

## Author Contributions

(I) Conception and design: FL, CZ, QX. (II) Administrative support: QX. (III) Provision of study materials or patients: XL, QX. (IV) Collection and assembly of data: FL, XL. (V) Data analysis and interpretation: FL, RL. (VI) Manuscript writing: FL; (VII) Final approval of manuscript: CZ, QX. All authors contributed to the article and approved the submitted version.

## Funding

National Natural Science Foundation of China Major Research Program Cultivation Project (91949113).

## Conflict of Interest

The authors declare that the research was conducted in the absence of any commercial or financial relationships that could be construed as a potential conflict of interest.

## Publisher’s Note

All claims expressed in this article are solely those of the authors and do not necessarily represent those of their affiliated organizations, or those of the publisher, the editors and the reviewers. Any product that may be evaluated in this article, or claim that may be made by its manufacturer, is not guaranteed or endorsed by the publisher.
