# Supplementary material for: Development and Validation of a Clinical Pregnancy Failure Prediction Model for Poor Ovarian Responders During IVF/ICSI
Source: Front Endocrinol (Lausanne). 2021 Aug 23;12:717288. doi: 10.3389/fendo.2021.717288 (PMC8419272; doi:10.3389/fendo.2021.717288)
Supplement: Supplementary file 1 [file DataSheet_1.docx]

**Supplementary 1 The decision curves of the prediction model**


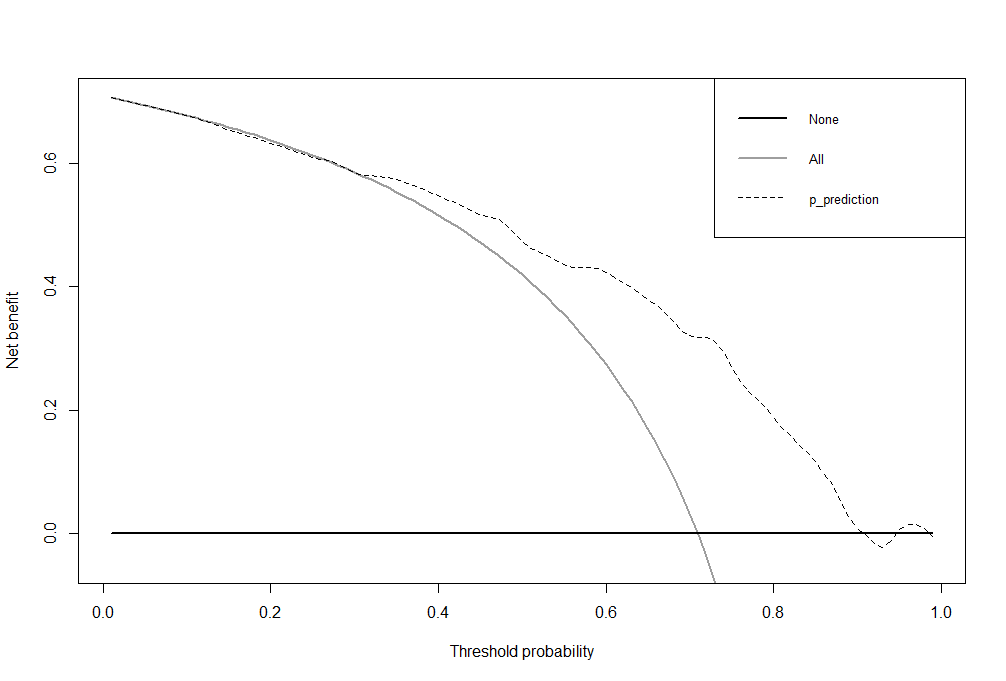


Draw the decision curve of the model with the net benefit as vertical axis and the threshold probability as horizontal axis. The solid black line represents the net benefit when all patients are considered as not developing outcome (failed conception). The solid grey line represents the net benefit when all patients are considered as developing outcome.

We created model A (before ET), model B (before the start of the cycle) and model C (on hCG day) to evaluate predictive powers at different stages.

The variables were selected by stepwise regression and then fit a model. Finally, as shown in Supplementary 2, six independent risk factors for clinical pregnancy included in the prediction model A. Four included in model B and five included in model C. In the training set, the AUCs of model A, B and C are 0.786 (95% CI: 0.710–0.861), 0.748(95% CI: 0.672–0.825) and 0.768(95% CI: 0.691–0.845), respectively (Figure 2A, Supplementary 3A, 3E). Model A showed the largest AUC which indicated that it had the best performance. Brier scores of model A, B and C are 0.160, 0.173 and 0.165, respectively (Figure 2B, Supplementary 3B, 3F). Model A had the lowest Brier score and showed the best power in prediction calibration. AIC (Akaike Information Criterion) was used to measure the goodness of model fit. The smaller the value, the better the model fit. The AIC of model A, B and C are 190.45, 196.32 and 192.49, respectively (Supplementary 2). Model A showed the smallest AIC with the best model fit.

In conclusion, model A had the best predictive performance, calibration power and model fit. So “before ET” may be the best predictive time point for clinical pregnancy failure based on our database. It is meaningful in clinical practice to advance the time point for predicting clinical outcomes. However, when the valid predictive factors increase as time goes on, the predictive performance of the model will be closer to reality. A large-scale and multi-central database is needed for advance researches.

**Supplementary 2 Comparison among 3 models**

| **Model** | **Variables** | **Regression coefficients** | **OR (95% CI)** | ***P* value** | **AUC** | **AIC** | **Brier** |
| --- | --- | --- | --- | --- | --- | --- | --- |
| **A** | **Age**＞**35 (years)** | **0.953** | **2.59 (1.24-5.47)** | **0.012** | **0.786** | **190.45** | **0.160** |
|  | **BMI**＞**24 (kg/m^2^)** | **1.169** | **3.22 (1.45-7.58)** | **0.005** |  |  |  |
|  | **Basal FSH**＞**10 (mIU/mL)** | **1.053** | **2.87 (1.28-6.75)** | **0.012** |  |  |  |
|  | **Basal E2**＞**60 (pg/mL)** | **0.902** | **2.47 (1.08-5.93)** | **0.036** |  |  |  |
|  | **Type B or C of endometrium** | **0.906** | **2.47 (1.18-5.24)** | **0.017** |  |  |  |
|  | **High quality embryos**＜**2 (n)** | **0.806** | **2.24 (1.02-4.96)** | **0.045** |  |  |  |
| **B** | **Age**＞**35 (years)** | **1.137** | **3.12** **(1.53-6.44)** | **0.002** | **0.748** | **196.32** | **0.173** |
|  | **BMI**＞**24 (kg/m^2^)** | **1.012** | **2.75 (1.30-6.13)** | **0.010** |  |  |  |
|  | **Basal FSH**＞**10 (mIU/mL)** | **1.063** | **2.90 (1.33-6.765)** | **0.009** |  |  |  |
|  | **Basal E2**＞**60 (pg/mL)** | **1.001** | **2.72 (1.23-6.41)** | **0.017** |  |  |  |
| **C** | **Age**＞**35 (years)** | **1.049** | **2.85 (1.38-5.96)** | **0.005** | **0.768** | **192.49** | **0.165** |
|  | **BMI**＞**24 (kg/m^2^)** | **1.020** | **2.77 (1.29-6.28)** | **0.011** |  |  |  |
|  | **Basal FSH**＞**10 (mIU/mL)** | **1.141** | **3.13 (1.41-7.33)** | **0.006** |  |  |  |
|  | **Basal E2**＞**60 (pg/mL)** | **0.925** | **2.75(1.12-6.01)** | **0.030** |  |  |  |
|  | **Type B or C of endometrium** | **0.895** | **2.44(1.18-5.13)** | **0.016** |  |  |  |

Model A: prediction before ET; model B: prediction before the start of IVF/ICSI cycle; model C: prediction on HCG day.

OR, odds ratio; CI, confidence interval.

AUC (The area under the curve) was used to evaluate the predictive accuracy. A higher AUC shows a better performance.

AIC (Akaike Information Criterion) was used to measure the goodness of model fit. The smaller the value, the better the model fit.

The lower the Brier score for a set of predictions, the better the prediction calibration.

**Supplementary 3 ROC curves and calibration plots of the training and validation set of model B and C.**

**
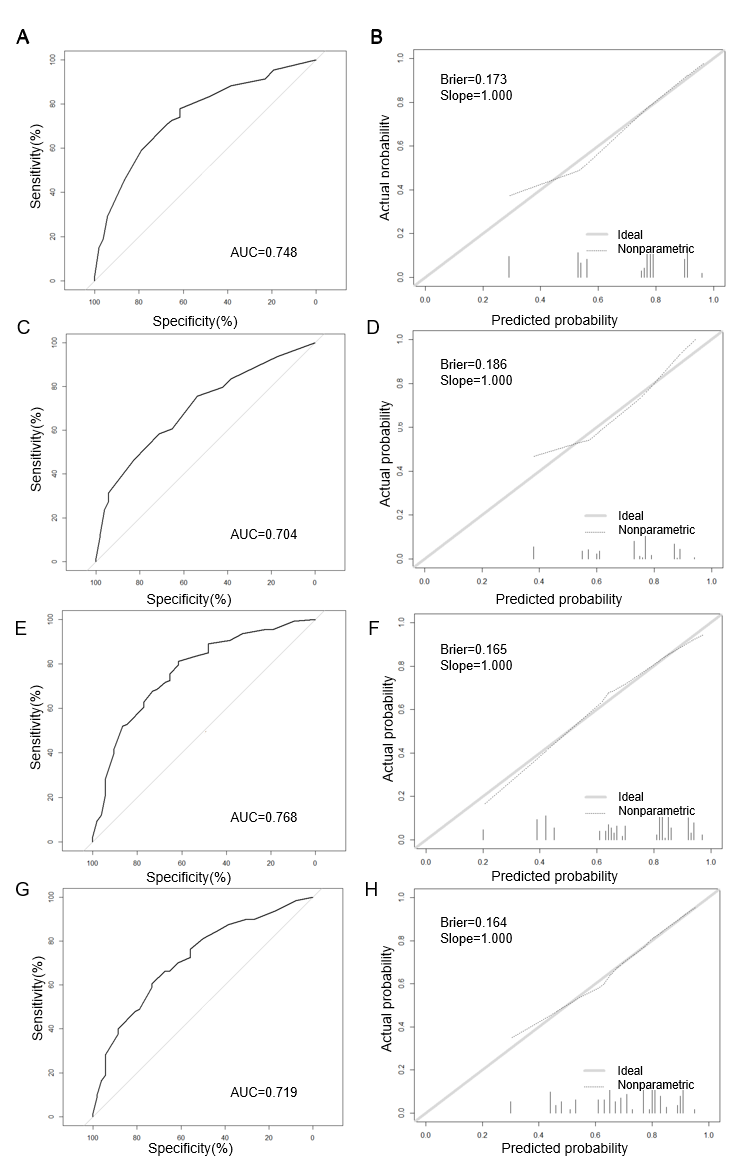
**

(A)AUC of model B in the training set is 0.748 (95% CI: 0.672–0.825). (B)Calibration curve of model B for training set (Brier=0.173, Slope=1.000). (C)AUC of model B in the validation set is 0.704(95% CI: 0.668–0.827). (D)Calibration curve of model B for validation set (Brier=0.186, Slope=1.000). (E)The AUC of model C in the training set is 0.768 (95% CI: 0.691–0.845). (F)Calibration curve of model C for training set (Brier=0.165, Slope=1.000). (G)AUC of model C in the validation set is 0.719 (95% CI: 0.638–0.800). (H)Calibration curve of model C for validation set (Brier=0.164, Slope=1.000). Calibration curves were used to evaluate the calibration of the model. The horizontal axis is the predicted probability provided by this model, and the vertical axis is the observed incidence of pregnancy failure. The ideal line with 45° slope represents a perfect prediction (the predicted probability equals the observed probability). The lower the Brier score for a set of predictions, the better the prediction calibration. When the slope was closer to 1.00, the prediction model had better calibration power.
